# A prospective cohort study of surgical treatment for back pain with degenerated discs; study protocol

**DOI:** 10.1186/1471-2474-6-24

**Published:** 2005-05-24

**Authors:** Richard A Deyo, Sohail K Mirza, Patrick J Heagerty, Judith A Turner, Brook I Martin

**Affiliations:** 1 Department of Medicine. Box 359736, 325 Ninth Ave., Seattle, Washington, 98104, USA; 2 Department of Health Sciences. Box 359736, 325 Ninth Ave., Seattle, Washington, 98104, USA; 3 Department of Orthopedic Surgery. Box 359798, 325 Ninth Ave., Seattle, WA 98104, USA; 4 Department of Biostatistics. University of Washington, Box 357232, 1959 NE Pacific Street, Seattle, Washington, 98195, USA; 5 Department of Psychiatry and Behavioral Sciences. University of Washington, Box 356560, 1959 NE Pacific Street, Seattle, Washington, 98195 USA; 6 Center for Cost and Outcomes Research. Box 359736, 325 Ninth Ave. Seattle, Washington, 98104, USA

## Abstract

**Background:**

The diagnosis of discogenic back pain often leads to spinal fusion surgery and may partly explain the recent rapid increase in lumbar fusion operations in the United States. Little is known about how patients undergoing lumbar fusion compare in preoperative physical and psychological function to patients who have degenerative discs, but receive only non-surgical care.

**Methods:**

Our group is implementing a multi-center prospective cohort study to compare patients with presumed discogenic pain who undergo lumbar fusion with those who have non-surgical care. We identify patients with predominant low back pain lasting at least six months, one or two-level disc degeneration confirmed by imaging, and a normal neurological exam. Patients are classified as surgical or non-surgical based on the treatment they receive during the six months following study enrollment.

**Results:**

Three hundred patients discogenic low back pain will be followed in a prospective cohort study for two years. The primary outcome measure is the Modified Roland-Morris Disability Questionnaire at 24-months. We also evaluate several other dimensions of outcome, including pain, functional status, psychological distress, general well-being, and role disability.

**Conclusion:**

The primary aim of this prospective cohort study is to better define the outcomes of lumbar fusion for discogenic back pain as it is practiced in the United States. We additionally aim to identify characteristics that result in better patient selection for surgery. Potential predictors include demographics, work and disability compensation status, initial symptom severity and duration, imaging results, functional status, and psychological distress.

## Background

Mechanical or chemical changes in degenerative intervertebral discs comprise a hypothesized cause of low back pain without sciatica or neurological deficits. This is commonly referred to as "discogenic" low back pain, and is distinct from a herniated disc causing sciatica. The diagnosis often leads to spinal fusion surgery and may partly explain the recent rapid increase in lumbar fusion operations in the United States (U.S.) [1,2].

A few randomized studies have examined outcomes of spinal fusion surgery compared to non-surgical treatment for discogenic pain; these suggest little or no advantage of surgery over carefully designed rehabilitation therapy [3-6]. In one study, an early advantage of surgery was lost with longer follow-up [4,7]. Major surgical complication rates have been as high as 19% [5,8]. Furthermore, the only randomized trials have been conducted in Europe, where dramatically lower surgery rates suggest a more selective practice style than in the U.S. [9]. Thus, more data are needed on safety and outcomes in routine care for discogenic back pain in the U.S.

Although the concept of a painful disc causing back pain has merit on theoretical grounds, mechanical and chemical changes develop in all vertebral discs with aging. Clinical studies have not firmly established diagnostic criteria that distinguish patients with painful discs from others with normal aging. The distinction is often based on lumbar discography, itself a controversial procedure troubled with high rates of false positive results [10-12]. Research also suggests that patients with psychological distress are more likely to report pain on discography than those without psychological distress [12,13]. Furthermore, clinical studies of patients with chronic back pain provide strong evidence that psychological distress is an important risk factor for having poor outcomes after spine surgery [14-16]. Some patients with psychological distress, therefore, may be preferentially treated with fusion, and paradoxically have poor outcomes. Even when patients are meticulously selected on the basis of discography and clinical screening, fusion results are often poor [12]; this challenges the concept that discography correctly identifies the source of pain and that spinal fusion corrects this problem. Little is known about how patients undergoing lumbar fusion compare in preoperative physical and psychological function to patients who have degenerative discs, but receive only non-surgical care. Finally, useful models to predict which patients will have a good response to surgical therapy have not been developed.

We implemented a prospective cohort study to compare patients with presumed discogenic pain who undergo lumbar fusion with those who have non-surgical care. The study is intended to address several questions. First, are there differences in preoperative physical and psychological function between those having surgical versus non-surgical treatment? For example, do patients undergoing fusion for discogenic pain have greater preoperative psychological distress than patients not undergoing fusion? Secondly, how do treatment outcomes, including symptoms, functional status, return to work and subsequent surgery, differ between the two groups? Do the results support the conventional wisdom that outcomes improve more after fusion than after non-surgical care? A final study objective is to identify predictors of favorable outcomes of both surgical and non-surgical care for discogenic pain. Are there characteristics that predict a good response to surgical therapy, but not to non-surgical therapy? Such knowledge could result in better patient selection for surgery. Potential predictor characteristics include demographics, work and disability compensation status, initial symptom severity and duration, imaging results, functional status, and psychological distress.

## Methods

We designed a multi-center prospective cohort study of patients with presumed discogenic back pain. We will use follow-up assessments over two years to compare health outcomes between those who receive a spinal fusion versus those who receive other treatment. Important secondary analyses will include comparisons of those who have elective surgery and those who do not in terms of preoperative physical and psychological function and identification of patient characteristics that predict outcomes. The study protocol was approved by the University of Washington (U.W.) Human Subjects Division and all participants provide written informed consent.

Patients are recruited from five orthopaedic clinics in the Puget Sound region of Washington State: the U.W. affiliated practice sites (U.W. Medical Center and Harborview Medical Center), Orthopaedics International (affiliated with Providence Medical Center and Evergreen Hospital), and Proliance Surgeons, Inc., P.S. (associated with Orthopedics Physician Associates and Swedish Hospital in Seattle). These practices include eleven surgeons who have referred patients to the study.

We identify patients with predominant low back pain as a symptom, one or two-level disc degeneration confirmed by imaging, and a normal neurological exam. Because surgery for discogenic pain is rarely considered as initial therapy, we required patients to have pain lasting for at least six months. We did not require discography for diagnosis. Additional inclusion and exclusion criteria are listed in Table 1. These eligibility criteria mimic those of the European randomized trials of surgery for disc for discogenic pain [4-6].

**Table 1:** Inclusion and exclusion criteria.

**Inclusion Criteria:**
Age greater than 20 years and less than 65 years
Back pain of greater than 6-month duration
Has telephone and willing to complete telephone interviews
Ability to communicate in English
Physician diagnosis of discogenic back pain
One or two level disc degeneration on MRI scan
**Exclusion Criteria:**
Leg pain greater than back pain
Pregnant
Instability on lumbar flexion-extension radiographs (if done)
Motor deficit on physical examination
Abnormal electrodiagnostic studies (if done)
Lumbar spinal stenosis requiring a laminectomy.
Prior lumbar fusion or multilevel laminectomy
Spondylolisthesis of more than 25% (>grade I)
Inflammatory spondyloarthropathy
Spinal malignancy or infection
Severe co-morbid illness that would contraindicate surgery or result in major functional decline
Developmental spine deformities or vertebral fractures
Disc protrusion or extrusion on imaging with either:
Nerve root compression or displacement, or pain radiating below the knee

Patients are classified as surgical or non-surgical based on the treatment they receive during the six months following study enrollment. We use both patient interview and medical record information to determine whether patients have had surgery.

Surgical details, including the type of procedure, levels operated, and surgical implants, are recorded from operative notes in the medical records. These details are important because there are several variations in surgical technique for spinal fusion for discogenic pain.

Non-surgical treatment of patients with chronic low back pain and degenerative discs may include physical therapy, exercise, cognitive-behavioral therapy, medication, injection, transcutaneous electrical nerve stimulation, alternative treatments such as acupuncture and spinal manipulation, and intradiscal electrothermal therapy (IDET). The treatments patients receive are recorded both at baseline and at follow-up interviews.

We conduct the baseline assessment in person and obtain the follow-up measures in telephone interviews at six, nine, twelve, and twenty-four months after enrollment. Also, after three months, we re-administer the baseline measures. For patients who have surgery late in the initial 6-month interval, this provides a more recent pre-surgical baseline for analysis.

We evaluate several dimensions of outcome, including pain, functional status, general well-being, and role disability. The measures have been previously validated and have been recommended for standardized outcome assessment in back pain trials [17,18]. Furthermore, most of these measures have been recommended by the American Academy of Orthopaedic Surgeons and the North American Spine Society [19]. These instruments were chosen with an eye towards brevity, established reliability and validity, ease of administration by telephone, and inclusion of a range of relevant outcomes.

The Modified Roland Scale is the primary outcome measure. The original Roland-Morris Disability Questionnaire [20] was derived from the 136-item Sickness Impact Profile (SIP) [21]. It was subsequently modified to select items from the SIP that would more likely detect change in patient status and to include attribution of limitations to leg pain as well as back pain [22]. This 23-item self-report measure of physical disability due to back and leg pain has established validity, reliability, and responsiveness to change [22]. Higher scores indicate a greater level of functional disability.

The SF-36 Health Survey, Version 2 [23] consists of eight scales (general health, physical functioning, role limitations due to physical problems, role limitations due to emotional problems, bodily pain, social function, mental health, and vitality) scored on a scale of 0 (worst health) to 100 (ideal health). The earlier version has been used to assess general health status among patients with variety of health conditions, including back pain [17,24,25].

Study participants also complete the Symptom Check List-90 (SCL-90) 12-item somatization and 13-item depression scales [26]. Subjects indicate whether they have each symptom using a 5-point scale ranging from "not at all" to "extremely". Higher scores indicate greater somatization or depression symptom severity.

The 13-item Pain Catastrophizing Scale [27] is used as both a predictor and a secondary outcome. The measure includes subscales that reflect three components of pain-related catastrophizing: helplessness, rumination, and magnification (e.g., fear that the pain will become worse). Previous research has consistently found substantial associations between pain-related catastrophizing and pain-related disability [28,29]. We are interested in learning whether pain-related catastrophizing is a risk factor for poor outcomes in patient with low back pain.

The baseline questionnaire also assesses demographics, pain (numerical rating scale, bothersomeness, other painful body sites), medical co-morbidity, back pain history, work status, and litigation/compensation issues. Problematic alcohol use is assessed by the first three items of the Alcohol Use Disorders Identification Test (AUDIT-C) [30]. The AUDIT-C has been shown to be a valid screening test for heavy drinking and/or active alcohol abuse or dependence [30].

Although imaging is required for enrollment, not all imaging studies are accessible to the researchers (e.g., those done at a distant site). However, a majority of study participants have magnetic resonance imaging (MRI) scans that are available to the investigators. These images are interpreted by a neuroradiologist without knowledge of the patient's clinical history. The key imaging findings were defined as in the Longitudinal Assessment of Imaging and Disability for the Back and include T2 signal, spondylolisthesis, endplate Modic changes, disc height loss, disc morphology, annular tears, facet changes, and central and foraminal stenosis [31].

All data are collected on paper forms, and then entered into a web-based data system requiring double entry to reduce transcription errors. Data checks to identify out-of-range answers, inconsistent responses, missing data, and response rates are performed on a monthly basis.

## Results

In the primary analysis, the modified Roland Disability scale at 24 months will be compared between the surgical and non-surgical treatment arms using regression techniques to adjust for important baseline characteristics.

Secondary analysis will include a comparison of pain, SF-36 scales, and psychological measures for the two treatment arms. To characterize time trends in the primary and secondary outcomes, we will use linear mixed models (or Generalized Estimating Equations in the case of categorical variables) to analyze the repeated measures obtained at all follow-up interviews. Finally, we will examine potential predictors of outcome, including baseline disability, psychological factors, and image findings. The goal will be to identify subgroups of patients who respond well to surgery but not to non-surgical therapy, or to non-surgical treatment but not surgery.

The Maine cohort study [25] provided crude estimates of possible differences between surgical and non-surgical patients on the Roland Scale, SF-36, and pain scales. Table 2 shows the estimated sample size needed to detect differences of various magnitudes between the surgical and non-surgical patients. Based on these estimates, our goal is to enroll 150 patients each in the spinal fusion arm and in the non-surgical treatment arm. Enrollment will proceed over two years. Based on estimates of eligible patient numbers, and our conservative estimate that 60% of eligible patients will enroll in the study, this will enable us to obtain the target enrollment.

**Table 2:** Estimated sample sizes per group, based on different outcome measures. For function and symptoms, standard deviations are from the Maine Lumbar Spine study, with 3-month follow-up (roughly equal proportions treated surgically and non-surgically). Return to work proportion is based on a study by the Washington State Department of Labor and Industries.

Category	Measure	Hypothesized difference between groups	N/group, 2-sided alpha = 0.05, power = 0.80
Functional Status	Difference in change in Roland score (0–23 scale, SD = 7.37)	2.5 points	136
		3.0 points	95
Symptoms	Improvement in pain (1 = gone, 5 = same, 7 = much worse) (SD = 1.841)	0.75 point	94
		1.0 point	53
Return to Work	Proportion currently employed, if lower proportion is 0.40	0.15	173
		0.20	97

## Discussion

In designing the study, we considered a randomized controlled trial (RCT), but chose an observational design for several reasons. First, the surgical treatments are already approved and in wide use, and physicians and patients often have strong preferences for either surgical or non-surgical care. Thus, genuine equipoise is rare among U.S. surgeons and patients. In fact, we attempted to enroll subjects in a pilot randomized trial and had no success. Second, randomized trials comparing surgery with non-surgical treatment have several features that are distinctly different from drug trials and result in serious limitations. If drugs cause side effects, they can be stopped and most ill effects will resolve. Surgery, however, has many irreversible features. Efforts to blind the treatment allocation require a sham surgical procedure, raising patient anxieties and ethical concerns. Without a sham surgical control, blinding is impossible. Unlike pills, which are essentially identical, no two surgical procedures are exactly the same. These features constrain the validity of surgical randomized trials in comparison to drug trials. Finally, we were interested in treatment outcomes as they occur in routine practice, rather than within the narrow constraints typically imposed in randomized trials. Thus, we chose to study treatment effectiveness in routine care, rather than efficacy under ideal circumstances. Though an RCT would provide the most valid data on efficacy, the prospective cohort design seemed substantially superior to uncontrolled case series, which remain the predominant study design in the surgical literature. Furthermore, when carefully designed, the results of cohort studies sometimes approximate the results of randomized trials [32,33]. The Maine Lumbar Spine Study [24,25], for example, was a prospective cohort study that yielded results concordant with those from randomized trials of discectomy [34].

## Conclusion

This study will contribute important new information on a highly controversial area of back pain treatment. Though it is not a randomized trial, we believe a rigorously designed and analyzed cohort study will improve our knowledge of both treatment effectiveness and safety in routine practice. The primary aim of this prospective cohort study is to better define the outcomes of lumbar fusion for discogenic back pain as it is practiced in the U.S. The results should help improve the selection criteria for surgical treatment, better define the prognosis after therapy, and improve our ability to match patients with optimal treatment approaches.

## Competing interests

The author(s) declare that they have no competing interests.

## Authors' contributions

RAD and SKM conceived of the study. RAD, BIM, SKM drafted the manuscript. PJH performed statistical analysis. All authors participated in the design of the study and coordination of the study. All authors read and approved the final manuscript.

## Pre-publication history

The pre-publication history for this paper can be accessed here:


